# Influence of Gender in Diabetes Mellitus and Its Complication

**DOI:** 10.3390/ijms23168850

**Published:** 2022-08-09

**Authors:** Tiziana Ciarambino, Pietro Crispino, Gaetano Leto, Erika Mastrolorenzo, Ombretta Para, Mauro Giordano

**Affiliations:** 1 Internal Medicine Department, Hospital of Marcianise, ASL Caserta, 81037 Caserta, Italy; 2 Emergency Department, Hospital of Latina, ASL Latina, 04100 Latina, Italy; 3 Department of Experimental Medicine, University La Sapienza Roma, 00185 Roma, Italy; 4 Emergency Department, Hospital of Careggi, University of Florence, 50121 Florence, Italy; 5 Internal Emergency Department, Hospital of Careggi, University of Florence, 50121 Florence, Italy; 6 Department of Medical Science, University of Campania, L. Vanvitelli, 81100 Naples, Italy

**Keywords:** diabetes mellitus, gender differences, complication

## Abstract

In medicine, there is growing evidence that gender differences are important and lead to variations in the pathophysiology and treatment of many diseases with traits that appear to be particularly relevant in influencing the outcomes of many morbid forms. Today, the inclusion of gender in biomedical research, to improve the scientific quality and scientific relevance of knowledge, of technology is an increasingly present element precisely due to the practical implications that derive from it. Gender differences describe the biological variability between women and men, which is, in turn, related to differences in the information contained in sex chromosomes, the specific gene expression of autosomes linked to sex, the different number and quality of sex hormones, and their different effects on systems and organs, without neglecting the fact that each of the sexes has different target organs on which these hormones act. Additionally, both genders undergo metabolic changes throughout their lives, and this is especially true for women who show more dramatic changes due to their role in reproduction. Gender differences are not only the result of our genetic makeup but are also mixed with socio-cultural habits, behaviors, and lifestyles, differences between women and men, exposure to specific environmental influences, different food and lifestyle styles or stress, or different attitude in compliance with treatments and disease prevention campaigns. Gender differences also affect behavior throughout life, and physical changes can have implications for lifestyle, social roles, and mental health. Therefore, determinism and therapeutic outcome in chronic diseases are influenced by a complex combination of biological and environmental factors, not forgetting that there are many interactions of social and biological factors in women and men. This review will address the role of gender differences in the management of various forms of diabetes and its complications considering the different biological functions of hormones, the difference in body composition, physiological differences in glucose and fat metabolism, also considering the role of the microbiota. intestinal, as well as the description of gestational diabetes linked to possible pathophysiological events typical of reproduction.

## 1. Background

In general, there are numerous reports from various countries that observe large differences in the ratio between the sexes in the determinism of diseases related to metabolism. The differences in biology, culture, lifestyle, environment, and socioeconomic status of the various populations under study obviously affect the differences between males and females in terms of predisposition, development, and clinical presentation of diabetes mellitus. Genetic inheritance and epigenetic mechanisms, nutritional factors, and sedentary lifestyle influence the risk and complications differently in both sexes, in consideration of the socio-cultural environment of the populations. Recently, it has been observed that diabetes mellitus can have some gender peculiarities and some data show that women have more years of disease on average than their male counterparts and have a higher body mass index (BMI). This seems to be related to the fact that sex hormones have a great impact on energy metabolism, body composition, vascular function, and inflammatory responses. Furthermore, in patients with type 1 diabetes, women show worse metabolic control over time, while in men, as the years of illness increase, other risk factors are associated, such as a higher incidence of uncontrolled or refractory arterial hypertension. standard therapies [1]. The sharp increase in type 2 diabetes mellitus and associated complications go hand in hand with the growing evidence of clinically important gender differences in the determinism of this form of diabetes. Thus, also in type 2 diabetes mellitus, it is highlighted that it is more frequently diagnosed in men who have a lower age and body mass index than in men; however, as with type 1 diabetes mellitus, the most important risk factor, which is obesity, is more common in women. Both biological and psychosocial factors are responsible for sex and gender differences in diabetes risk and outcome. Overall, psychosocial stress appears to have a greater impact on women than on men. Gender differences are equally important in the development, awareness, presentation, diagnosis, and therapy, as well as in the prevention of the lifestyle-associated disease of patients with diabetes mellitus. It is also true that awareness of the disease and the severity of its complications may not only depend on gender in the strict sense but is also related to the experience of the man or woman and, therefore, is dependent on the level of education, income, and quality of services, and social and lifestyle support. Therefore, it is almost impossible to always make a clear distinction between gender influences and exogenous factors or differences in everyday life because these complex factors are interconnected and interact with each other throughout life. Based on all these facts, in this review, gender differences will be described to indicate the main biological differences and very generally to describe the predominant psychosocial influences, but not universally concerning the reality present in the world population of individuals affected by diabetes. The great impact of psychosocial risk factors in addition to biological ones is the considerable regional differences and the prevalence in the various territories of the various forms of diabetes in adult men and women. Most patients with diabetes live in low-income countries, but prevalence rates in high-income countries are comparable to those in developing countries and we consider the population with the lowest socioeconomic level. This mainly depends on the diet of these populations shifted to greater consumption of traditionally low-cost foods that have a high content of simple or complex sugars and this goes hand in hand with the considerable regional differences found in the increase in the phenomenon of obesity [2]. In addition, these estimates may also be influenced by the fact that women in many parts of the world often suffer from inequality in access to primary and secondary screening controls and more generally in access to health care. In women, diabetes mellitus appears to be less controlled considering each metabolic parameter, especially given the fact that they tend to have lower insulin sensitivity than their male counterparts, resulting in greater use of insulin units to maintain optimal glycemic values and compliant with therapeutic goals [2]. These differences in glucose homeostasis between the two genders are to be found above all in the pre-diabetic state where there is a reduced fasting glycemia, especially while women are more prone to develop a reduced glucose tolerance in response to a meal or glucose load. [2]. In addition, other evidence comes from the clustering disease of drug treatment of diabetes mellitus, in which women are at higher risk of suffering from hypoglycemia using insulin, of urinary and genital tract infections using glycosuric drugs such as glyphozines, and following the use of thiazolidinediones, show an increased risk of postmenopausal bone fractures [2]. After what has been said, the genetic background, lifestyle, and environmental aspects contribute greatly to the complexity of managing the diabetic patient and therefore, this review will provide some clarification on the gender differences in diabetes mellitus.

## 2. Methods

In this narrative review, we have included clinical studies published by Pubmed up to 30 May 2022. The keywords used were diabetes, comorbidities, and gender differences. All articles and clinical publications published by Pubmed were studied by two authors. Studies written in languages other than English were excluded. Two authors (PC, TC) reviewed all articles, and all studies were qualitatively analyzed. The objective of this review is to provide the necessary knowledge for decision support in the field of diabetic pathology to promote robust approaches to managing disease and its complications in functional gender. Despite the difficulty in finding scientific studies that emphasize the role of gender in diabetic pathology and its complications, the rationale of reviewing this pathology from a gender perspective remains an important function of health care, as a lack of initial knowledge of this phenomenon can lead to loss of opportunities for investigation and treatment and will certainly increase the level of adequate knowledge in the future with consequent positive effects on the complete management of diabetes mellitus. We conducted a comprehensive systematic search.

## 3. Types of Diabetes and Gender

### 3.1. Type 1 Diabetes

In type 1 diabetes, the pancreas is unable to produce insulin due to the destruction of the beta-cells that can produce this hormone. It affects about 3–5% of people with diabetes and generally occurs in childhood or adolescence but can also occur in adults. Type 1 diabetes is therefore characterized by beta-cell destruction, on an autoimmune or idiopathic basis, which leads to absolute insulin deficiency. The cause of type 1 diabetes is unknown, but it is now known that at the base of the disease there is a “sabotage” by the immune system against the cells that produce insulin: the disease manifests itself in the presence of direct antibodies in the blood against antigens present in the cells that produce insulin. It is, for this reason, that type 1 diabetes is classified among the so-called “autoimmune” diseases. The damage that the immune system causes to the cells that produce insulin is believed to be linked to hereditary factors and/or environmental factors (including diet, lifestyle, and contact with specific viruses). Type 1 diabetes is the only common autoimmune disease not characterized by female predominance [2]. The pubertal period is associated with a reduced incidence of Type 1 diabetes in girls who maintain stronger residual β-cell function than boys [3]. This suggests that female gonadal hormones transiently protect against type 1 diabetes and in general young adolescents with reduced serum estrogen activity develop this form of diabetes [4,5]. In experimental models obtained from rats, it was observed that the use of estradiol protects pancreatic islets from multiple metabolic and pro-inflammatory lesions in vivo by restoring the immunomodulatory functions of natural killer cells [6,7]. In contrast to previous studies that have shown no seasonality in the incidence of type 1 diabetes, a Japanese study showed a higher incidence in women aged 0–19 years. These results, including the interaction we showed between age ranges and seasons, may indicate that childhood-onset type 1 diabetes and adult type 1 diabetes have different modes of onset [8]. Type 1 diabetes is diverse in its genetics, environmental influences, immunology, metabolic characteristics, and clinical course. The heterogeneity of type 1 diabetes stems from its polygenic nature, with interactions of multiple genes with environmental factors. Recent advances in type 1 diabetes genetic knowledge and methods have facilitated genotype–phenotype correlations. Age is a marker of heterogeneity in type 1 diabetes. Younger age is associated with higher risk and rate of progression through the stages of the disease, as well as distinct histological characteristics, immunological patterns, and genetic influences. In addition, clinical heterogeneity in type 1 diabetes is reflected by differences in the risk of complications, which is related to a wide array of factors, from hyperglycemia and longer diabetes duration, to race, ethnicity, and socioeconomic factors [9].

#### Prediabetes

Prediabetes is a serious health condition that occurs when blood sugar levels are higher than normal, but not high enough to diagnose type 2 diabetes. The prevalence of prediabetes differs between the sexes, giving rise to clinical implications: Men develop fasting blood glucose more often, while women more often show high post-meal blood glucose levels. The increase in fasting glucose is characterized by increased hepatic glucose production and reduced early insulin secretion, while postprandial hyperglycemia is mainly due to peripheral insulin resistance [10]. Post-prandial hyperglycemia can better predict progression to diabetes and is burdened by a greater risk of mortality since it is more strictly the cause of an increase in cardiovascular risk. This underlines the importance of performing oral glucose tolerance tests for screening especially in women if an initial alteration of glucose metabolism is suspected.

### 3.2. Type 2 Diabetes

The prevalence of type 2 diabetes is also characterized by a gender difference. Overall, the global prevalence of diabetes is higher in men, but there are more women with type 2 diabetes than men [2]. The gender difference in the prevalence of diabetes is reversed according to the stage of reproductive life: that is, there are more diabetic men before puberty, while there are more diabetic women after the age of menopause and in old age. The combined effect of more elderly women than men in most populations and the increasing prevalence of diabetes with age is the explanation given for this observation. This is an important observation because, as discussed above, women show more impaired glucose tolerance after a meal. Therefore, considering only fasting blood glucose as a screening and with the exclusion of post-prandial blood glucose values in calculating the prevalence of diabetes in women one could risk underestimating a much more evident phenomenon [11]. Faced with a greater prevalence of type 2 diabetes in women, it is important to underline that the forms of diabetes complicated by ketosis or diabetic ketoacidosis are encountered more in males [11]. The protection that women enjoy against ketosis and ketoacidosis only fails if there is a hypoestrogenic state or a prolonged anovulatory condition [11]. It is also interesting to note that the alterations in glucose homeostasis after meals are also associated with an increase in visceral adipose tissue and the presence of the latter does nothing but amplify insulin resistance in women, influencing the control of the values more. glycemic levels, on glycated hemoglobin, on average glycemia over 24 h, and increasing the therapeutic need for oral hypoglycemic agents in reaching the desired glycemic target [11]. Vitamin D can also directly stimulate insulin receptor expression, thereby improving glucose transport in human cells [12]. A significant interaction between sex and vitamin D was found prior to the sex-stratified analysis. In a population-specific cross-sectional study, a low level of vitamin 25(OH)D3 was found in middle-aged Caucasians, independently associated with type 2 diabetes in women but not in men [12]. The likelihood of having newly diagnosed or known diabetes has more than doubled in women. Current type 2 diabetes guidelines to intensify therapy after metformin are largely based on potential added benefits (e.g., weight reduction) or increased risk of side effects (e.g., hypoglycemia). The reduction in MACE with therapy with sodium-glucose co-transport-2 inhibitors (SGLT-2Is) appear to be significantly less in women with diabetes vs. men, while glucagon-like peptide-1 receptor agonists (GLP-1Ras) confer a similar reduction in major adverse cardiac events, irrespective of the gender [13,14].

### 3.3. Gestational Diabetes Mellitus

Gestational diabetes mellitus is a pathological entity in which the state of pregnancy promotes or worsens greater insulin resistance in predisposed women or because they are already overweight/obese by altering glucose homeostasis after meals. This form of diabetes does not always limit itself or tends to disappear after pregnancy but where it occurs it represents an important independent and strong female risk factor for the possible onset of a form of type 2 diabetes [15]. As mentioned, gestational diabetes occurs especially in women already affected by a certain level of insulin resistance such as obese or overweight, however, women of normal weight can also be susceptible to this form of diabetes due to genetic traits. which would appear in conjunction with the physiological increase in insulin resistance during pregnancy. Despite the possibility of undertaking intervention strategies against gestational diabetes, over 70% of women with it develop a form of type 2 diabetes mellitus [16]. Furthermore, it is known that gestational diabetes is more associated with adverse pregnancy outcomes that not only affect mothers but also affect the newborn [15,16]. For the newborn, there may be an increase in birth weight, an increased risk of hypoglycemia in the neonatal period, an increased risk of obesity in childhood or adolescence, and an increased risk of type 2 diabetes in adulthood compared to the children of a mother who did not have gestational diabetes. Newborns are at risk of respiratory distress, hypoglycemia, hypocalcemia, hyperbilirubinemia, polycythemia, and hyperviscosity. Furthermore, recent studies report that a pregnant woman with a male fetus has a higher risk of developing gestational diabetes suggesting that the segregation of sex chromosomes during fecundation affects to some extent the metabolic fate of the parturient in the period of gestation [17,18,19]. Since the underlying mechanism is unclear, the authors hypothesized several pathophysiological causes of these sex differences in β-cell function in mothers. This could be related to the actions of the Y chromosome on sex-specific changes in placental-derived hormones, as placental lactogen and prolactin or other proteins are involved in the expansion of β cell mass [18]. Interestingly, the placenta in particular exhibits many sex-specific alterations, including epigenetic mechanisms, which could really have a huge impact on complications during and after pregnancy. These have recently been examined in detail elsewhere [18,19]. Collectively, these new findings highlight the impact of fetal sex on maternal glucose metabolism. The constant interaction between fetus and mother, with potential future negative health impacts on both, clearly demonstrates critical health neglect not only in the field of glucose metabolism, but also in the field of gestational diabetes mellitus and fetal sex.

### 3.4. Menopause and Type 2 Diabetes in Women

The diabetic effect of menopausal estrogen deficiency in women can be considered a form of gender difference between the two sexes. The exact effect of menopause on impaired glucose metabolism in women, regardless of aging, has been studied by various authors who agree that early menopause (before the age of 40) is associated with an increased risk of type 2 diabetes compared to the menopause that occurs after the age of 50 and that the cause of this is precisely the lack of estrogen since women who have undergone surgical oophorectomy also have the same increased risk of diabetes [20,21]. The lack of estrogen due to menopause contributes to the development of type 2 diabetes with three different mechanisms which include the alteration of insulin secretion by the beta cells of the pancreas, the reduced sensitivity to insulin by the organs and tissues targeted, and increased sensitivity to glucose by the main organs of diabetes-related pathology [21]. However, it should be emphasized that the protective action of estrogens on the onset of diabetes occurs within a certain physiological range since it has been observed that if circulating estrogens are physiologically increased or if they are used synthetically in the form of oral contraceptives, insulin resistance develops. [20,21].

## 4. Gender and Metabolic Conditions Associated with Diabetes

### 4.1. Anthropometric Parameters

In women, aging and the transition from fertile life to menopause, with the loss of estrogen production, is associated with changes in body shape and a preferential increase in abdominal fat and perivisceral adiposity [22]. The lack of estrogen and increasing insulin resistance means that diabetic women have a stronger association of obesity than diabetic men [23]. Sex differences in body composition and fat deposition clearly contribute to the diversification of diabetes risk between genders [21]. Increased leg fat has been found to be associated with reduced cardiometabolic risk, especially in women, while increased trunk fat is generally related to clustering of cardiometabolic risk factors in cross-sectional population-based studies [22,23]. Recent studies have used the waist–call ratio as the most effective index for evaluating the proportions between abdominal fat and leg muscle mass and have shown that a favorable ratio to the former is an independent predictor of cardiovascular disease, metabolic disease, steatosis, and hepatic fibrosis. In line with this, women have a more noticeable increase in the waist–call ratio which becomes more and more noticeable with increasing age than men. This also translates into a greater cardiometabolic risk in women, in terms of glycemic and lipid abnormalities compared to males [23] and a cardiovascular risk in women that becomes increasingly higher over the years.

### 4.2. Brown Adipose Tissue

A high amount of brown adipose tissue is associated with reduced blood sugar levels in humans, suggesting that brown adipose tissue, which is metabolically more active because it carries out a greater turnover of fatty acids, also has a protective effect against diabetes. However, the relationship between the consumption of glucose by brown adipose tissue and systemic glycemic homeostasis is not so simple to define. Women have a much higher prevalence and activity of this tissue [24]. It was observed only in mice, the activity of brown adipose tissue is regulated by the growth factor for fibroblasts which in turn is positively regulated by estrogen [25]. Therefore, especially in women, a greater presence of brown adipose tissue could help reduce the risk of diabetes.

#### 4.2.1. Adipokines

Adipokines are all molecules produced and secreted by adipose tissue with autocrine, paracrine, or endocrine functions. Adipokines can be hormones, cytokines, chemokines, regulators of lipid metabolism, regulators of glucose homeostasis, growth factors, proteins of the alternative complement system, proteins involved in vascular homeostasis and pressure regulators, proteins involved in angiogenesis, acute and stress response inflammatory proteins, or components of the extracellular matrix. Some gender differences were also highlighted in the expression of these molecules [26]. Leptin and adiponectin are important in regulating satiety, food intake, and energy expenditure and can also influence the main mechanisms that determine peripheral insulin resistance [26]. In general, women have higher leptin and adiponectin levels than men [26]. The increase in plasma leptin is strongly correlated with the increased risk of diabetes in males [26]. Women show an upregulation of adiponectin and its receptor expression in abdominal adipose tissue which coincides with a lower risk of diabetes and cardiovascular disease. In obese and diabetic subjects, an inverse correlation is observed between plasma adiponectin levels and insulin sensitivity, and this relationship is more frequently observed in women than in men [26].

#### 4.2.2. Hepatic Steatosis

The liver is involved in various metabolic pathways, of which the control of glucose production, the destruction of insulin, and the synthesis and sorting of fats are of great importance. Fatty liver disease increases the risk of atherosclerosis and type 2 diabetes [27]. Women usually show less tendency to develop fatty liver disease than men and appear to be protected against estrogens in the premenopausal age with a marked degree, described in older women [28]. Furthermore, a sex-specific association between hepatic transaminase levels and insulin sensitivity has been described [29].

### 4.3. Sex Hormones

Sex hormone-binding globulin (SHBG) is a protein produced in the liver that is capable of binding testosterone, dihydrotestosterone, and estradiol (estrogen) and transporting them in an inactive form into the bloodstream. Elevated SHBG levels may indicate a polymorphism-mediated risk of diabetes that the SHBG gene may undergo [30]. In general, women tend to have higher SHBG levels than men while reduced SHBG concentrations in women may be associated with higher diabetes risk [30].

## 5. Gender Medicine for Diabetes and General Risk Factors

### 5.1. Social and Environmental Risk Factors

Modifiable social factors, such as low level of education, employment, and income, contribute to a large extent to unhealthy lifestyle behaviors and social disparities and, therefore, are related to a higher risk of obesity and type 2 diabetes, particularly in women [31]. Socioeconomic status understood as the level of education, social position, and income achieved, is inversely associated with the prevalence of obesity and type 2 diabetes in developed countries [31]. A strong inverse association between employment and newly detected diabetes was presented only in women [32]. The risk of diabetes is closely related to indicators such as lower household income and food insecurity and is higher in women but not in men [33]. In conclusion, the studies state that women seem more sensitive to socio-environmental predictors and context, such as education, income, and employment, for the future development of diabetes risk.

### 5.2. Psychosocial Stress and Sleep Disturbances

Discrimination of gender roles further increases environmental psychosocial stress, as well as stress responses, especially in women. Females appear to be more vulnerable to the adverse effects of the cardiometabolic impact of psychosocial stress, work stress, and sleep disturbances, as well as partly due to unhealthy behavior [34,35]. However, altogether the results are controversial [36]. Greater amounts of unpaid housework and family responsibilities may contribute to feelings of mixed demands and sustained stress levels in women, even in matched highly educated employee groups [37]. In a sex-specific meta-analysis of epidemiological studies, it was shown that women of all ages have a 40% higher risk of suffering from insomnia [38]. In turn, sleep loss, short sleep duration, and reduced sleep quality were related to obesity and even more strongly to insulin resistance-related (impaired glucose metabolism) [39]. In a meta-analysis, both short sleep (<5 h) and difficulty initiating or maintaining sleep were associated with higher diabetes risk. However, comparable effect estimates were observed in both sexes after stratification by sex [39]. In a smaller prospective study looking for sex differences as a primary outcome, sleep deprivation led to increased food and fat intake; however, males were more susceptible to weight gain based on a higher daily calorie intake, especially during the night [36]. The results of a meta-analysis of observational studies, with subgroups, analyzed by gender, showed that shift work was associated with an increased risk of diabetes in men [36]. In population-based cohort and occupational cohort studies, work strain has overall implied a higher diabetes risk in women, especially in those who perceive a combination of lack of control and high job demand, as well as low emotional support [40]. The controversial findings of sex and gender differences in the work-stress–diabetes risk relationship can be explained by differences in tolerance to inter-individual shift work, in the selection of occupational groups, and in the specific definitions of work stress in the studies, as well as differences in opportunities. for the recovery from work stress between men and women. Taken together, these studies suggest that mismatches between the circadian clock and social rhythms and between sex-dependent biological factors such as body composition and gender-dependent social time affect the pathogenesis of diabetes in men and women.

### 5.3. Lifestyle and Nutrition

There are substantial sex differences in health behavior, nutrition, and physical activity, which are closely associated with the risk of type 2 diabetes. According to sex-stratified health research data, women are overall more inactive but engage more in a healthy diet by consuming more fruit and vegetables and less meat [40]. The rapid economic development and the simultaneous increase in the consumption of food derived from fast food led to increased consumption of carbonated drinks, which contributes to the type 2 diabetes epidemic regardless of adiposity. In a prospective cohort study with separate analysis for men and women, only women showed an increased risk of incident type 2 diabetes over 10 years, with a doubled risk in women with daily soft drink consumption compared with non-alcoholic drinks. consumers [41]. Overall, only a slightly higher number of deaths, however, have been found to have a minimally lower percentage of deaths attributable to sugar-sweetened beverages for diabetes in women than in men [42]. In several observational studies, it has been shown that moderate alcohol consumption is associated with a lower risk of type 2 diabetes. In fact, another meta-analysis based on intervention studies showed that moderate alcohol consumption improves glycated hemoglobin. in both sexes but tends to improve insulin sensitivity only in women [43]. A cross-sectional analysis of the Nurses’ Health Study indicated that frequent alcohol intake was independently related to higher levels of endogenous estradiol and that estradiol alone influenced the protective association between alcohol consumption and diabetes risk in patients, postmenopausal women [44]. Further research is needed to elucidate the sex-specific dose–response relationships between alcohol consumption and type 2 diabetes risk and the exact underlying mechanisms. The role of smoking and smoking has changed substantially between men and women. Over the past decade, it has particularly increased in young women, potentially contributing to a higher incidence of smoking-related diabetes in women in the future [45]. Furthermore, a meta-analysis showed that the relative risk of myocardial infarction, a major and frequent complication in diabetic subjects, conferred by smoking appears to be 25% higher in women than in men [46]. A meta-analysis of cohort studies with subgroup analyses by gender, both active and passive smoking is correlated with a higher risk of developing type 2 diabetes in both men and women with no known prominent sex differences [46].

## 6. Complications of Diabetes and Gender

### 6.1. Cardiovascular Complications

In newly diagnosed diabetic subjects without clinical cardiovascular disease, increased atherosclerosis, and increased thickness of the intima-media in the carotid arteries were found [47]. In general, the male sex, in addition to age, was more associated with carotid plaques than the female sex. However, subgroup analysis revealed that, unlike male groups, carotid atherosclerosis was more prevalent in newly diagnosed diabetic women than in non-diabetic control women. These data confirm that in the presence of diabetes, the protective effect that young women have is attenuated with a consequent increase in the risk of cardiovascular events to a greater extent than in males [48]. Diabetes in association with hypertension, low physical activity, and high alcohol intake were stronger predictors of myocardial infarction in women than in men [49,50]. Furthermore, diabetes seems to compromise the microcirculation more by altering the endothelial response in women in a more dramatic way than in males, canceling the beneficial effects of estrogens [51]. Considering the specific results broken down by sex, a higher atherothrombotic risk is highlighted for women with type 2 diabetes mellitus linked to greater prothrombotic activity, and greater fibrin activity [52]. Another study investigated the effect of diabetes on the development of collateral vessels after chronic total occlusion by observing that in affected females there was poor angiogenesis in response to thrombotic insult [52]. Postmenopausal women are at a higher risk of ischemic stroke than men of comparable age. Furthermore, the frequency of stroke recurrence in the years following the first event is higher in diabetic women regardless of age. Considering the gender ratio, the risk of fatal coronary heart disease or stroke is higher in women [49,50].

### 6.2. Heart Failure

Obesity, hypertension, and diabetes are all important and independent risk factors for heart failure and can cause deterioration of myocardial function and metabolism more in women than in men [53]. Males tend to suffer from heart failure more often at a young age with a greater tendency to develop dilated cardiomyopathy while women tend to develop hypertrophic cardiomyopathy more often with diastolic heart failure and preserved ejection fraction [54]. The main differences in gender and in the presentation of heart disease associated with myocardial pump failure are to be found in the different adrenergic responses to physical activity with higher effects in women causing differences in lipid metabolism and contributing to heart muscle hypertrophy [55].

### 6.3. Diabetic Foot

Men develop diabetic foot syndrome at an early age and more frequently experience lower limb amputations [56,57]. Age, body mass index (BMI), and systolic blood pressure in men, while age, uric acid levels, and insulin therapy were independent risk factors for disease progression in women. Male sex, peripheral arterial disease, and renal failure are predictors of long-term death [58].

### 6.4. Diabetic Retinopathy

Diabetic retinopathy does not show a clear gender prevalence. Furthermore, the evidence available to date on the potential pathophysiological bases capable of explaining the identified gender differences is very limited.

### 6.5. Diabetic Nephropathy

A higher prevalence of vascular and renal damage in women was observed in insulin-resistant patients, with a greater increase in the intima-media thickness, a greater number of vascular plaques, and a reduction in blood flow at the level of the afferent artery [59]. Despite this, however, men show a more rapid progression of diabetic nephropathy and more often undergo dialysis therapy [60]. However, diabetic women have a higher mortality risk than diabetic men during chronic dialysis treatment [61]. The death event can occur for various causes, including cardiovascular ones, comorbidities related to old age, and plows always characterized by a progressive deterioration due to worsening of glycemic control. Underlying this has been recognized increased oxidative stress in diabetic women with end-stage renal disease. Sex hormones may be a key to explaining these differences between men and women, generally suggesting that women are protected from estrogen, hindering the progression of non-diabetic kidney disease at least before menopause [62]. In older adults with type 2 diabetes, long-term effects of a moderate protein diet (MPD) regimen are associated with a significant decline of renal function, proteinuria, low-grade inflammation, and oxidative stress without a change in fat-free mass [63].

Giordano et al., reported that in elderly Type 2 diabetic patients, long term effects of LPD 6/7 regimen in comparison to LPD 7/7 are associated with a similar decline in CrCl, but with decreased depressive symptoms and a better quality of life [64]. No data are reported on the long-term effects of a low protein diet (LPD) by gender on depressive symptoms and the quality of life in elderly Type 2 diabetic. 

### 6.6. Diabetic Neuropathy

Diabetic neuropathy, one of the most common and disabling chronic complications of the disease, is characterized by a broad spectrum of functional and structural alterations of the peripheral nerves that affect a proteiform clinical picture, of which distal symmetrical peripheral neuropathy is the most common [65]. Women show an increased risk of developing painful symptoms of neuropathy, especially of an algogenic and nociceptive nature, and more frequent symptoms of neuropathy such as paresthesia and loss of sensation in the feet [66].

### 6.7. Diabetic Gastroparesis

Many studies have shown a higher prevalence of gastroparesis in women than in men, other studies have not highlighted gender differences. In some studies, it has been pointed out that even in diabetic subjects without gastroparesis, gastric emptying is slower in women than in men [67,68]. It is known that glucose metabolism is influenced by gastric emptying and intestinal glucose absorption and the gender difference could be related to the way in which higher glucose is absorbed in women, possibly due to slower gastric emptying [69,70].

### 6.8. Mental Disorders

Anxiety, eating disorders, and depression are more common in people with diabetes, especially women [71]. Diabetic men appear to live more effectively with their disease, displaying a lower prevalence of depression and anxiety, more problem-oriented and resolution strategies, a better health-related quality of life, and positive well-being [72].

### 6.9. Sexual Function and Reproduction

Diabetic males and females are prone to suffer from sexual dysfunction and reproductive problems, which appear to be underestimated in clinical practice. Pre-pregnancy care in diabetic women of childbearing age is still unsatisfactory, due to steadily increasing rates of severe perinatal complications, including excessive malformation rates and higher mortality [73].

## 7. Conclusions

Gender medicine plays a fundamental role in the genesis of diabetes and in the development of various complications. Sex hormones play a role, at least in part, in these sex differences by regulating glucose homeostasis, insulin secretion, and action as well as influencing the progression of diabetes and various complications. Knowledge of the interactions between the endocrine regulation system and homeostasis is essential to promote the development of therapeutic alternatives for diabetes based on gender differences. Newer glucose-lowering agents used with metformin were associated with a lower risk of major adverse cardiovascular events. This beneficial effect was more pronounced in women than in men, especially for GLP-1RA users. Newer agents were also associated with a lower risk of adverse events, with no clear sex–drug interactions. The cholesterol absorption inhibitor ezetimibe and PCSK9 inhibitors have also been shown to lower risk in patients with diabetes. Recently, the eicosapentaenoic (EPA) only n-3 fatty acid, icosapent ethyl, has also shown benefit for cardiovascular risk reduction in patients with diabetes. To date, no agents targeting HDL increase have shown cardiovascular benefit in patients on background statin therapy. Difference in response to these drugs may be related to pharmacokinetic and pharmacodynamic differences based on gender. Women have a higher percentage of body fat and lower plasma volume with less organ blood flow. Increased body fat content explains the faster onset and prolonged duration of action and higher volume of distribution of lipophilic drugs, while the volume of distribution of hydrophilic drugs is smaller, producing higher initial plasma levels and greater effects when compared with males. Hepatic drug clearance is a function of cardiac output and liver blood flow, which are lower in women, while hepatic enzyme activity involved in phase I and II reactions, and transporters exhibit sex-specific differences.

## Data Availability

Not applicable.
